# Identification and Evaluation of Hub Long Non-Coding RNAs and mRNAs in PM2.5-Induced Lung Cell Injury

**DOI:** 10.3390/ijms26030911

**Published:** 2025-01-22

**Authors:** Jing Sui, Yanni Zhang, Linjie Zhang, Hui Xia

**Affiliations:** 1 Research Institute for Environment and Health, Nanjing University of Information Science and Technology, Nanjing 210044, China; suijingseu@163.com (J.S.); zhangyannijs@163.com (Y.Z.); zhanglinjieedu@163.com (L.Z.); 2 Key Laboratory of Environmental Medicine Engineering, Ministry of Education, School of Public Health, Southeast University, Nanjing 210009, China

**Keywords:** fine particulate matter, weighted gene co-expression network analysis, long non-coding RNAs, lung injury

## Abstract

Exposure to air pollution, especially fine particulate matter (PM2.5), is closely linked to various adverse health effects, particularly in the respiratory system. The present study was designed to investigate the lncRNA–mRNA interactions in PM2.5-induced lung cell injury using weighted gene co-expression network analysis (WGCNA). We downloaded the gene expression data of GSE138870 from the Gene Expression Omnibus (GEO) database and screened for differentially expressed lncRNAs and mRNAs. We constructed co-expression modules with WGCNA. Furthermore, functional enrichment analysis was also performed. We also constructed lncRNA–mRNA co-expression networks and lncRNA–mRNA-pathway networks to identify key regulatory relationships. The results revealed several modules significantly correlated with PM2.5-induced lung injury, such as the turquoise and blue modules. Genes within these modules were enriched in pathways related to signal transduction, metabolism, and cancer. Hub lncRNAs in the turquoise module, including LOC100129034 and CROCCP2, were found to be co-expressed with mRNAs involved in apoptosis and proliferation regulation. In the blue module, lnc-CLVS2-2 and GARS1-DT were connected to genes related to cell migration, invasion, and lung injury. These findings contribute novel perspectives to the molecular mechanisms involved in PM2.5-induced lung injury and suggest that WGCNA could be a valuable tool for predicting and understanding this disease process.

## 1. Introduction

Exposure to air pollution significantly impacts human health, correlating with several health conditions such as immune, respiratory, cardiovascular, nervous, and metabolic diseases [1,2,3,4]. In 2013, IARC listed air pollutants as Class I human carcinogens [5]. Functionally, the lungs are linked directly with the external environment via the upper respiratory tract, with a large respiratory surface area. Research consistently indicates that the respiratory system, as the initial barrier against external environmental threats, shows heightened susceptibility to pollutants, including particulate matter, volatile organic compounds, sulfur dioxide, and nitrogen dioxide [6].

Fine particulate matter (PM2.5) is globally recognized as a major air pollutant that poses a serious threat to public health. Research has indicated that PM2.5 pollution has become the fourth leading risk factor for health, in addition to high blood pressure, smoking, and poor diet [7]. PM2.5, with an aerodynamic diameter of ≤2.5 μm, is susceptible to deposition in the alveoli, inducing oxidative stress, inflammation, epithelial barrier dysfunction, and epigenetic changes [8]. PM2.5 exposure has been correlated with a variety of adverse pulmonary outcomes. Short-term exposure to PM2.5 is known to impair pulmonary expiratory flow rates and induce significant lung inflammation [9,10]. Li et al. [11] demonstrated that short-term exposure to PM2.5 induces inflammation and epithelial alterations, which were subsequently resolved, with macrophages, neutrophils, and type II alveolar epithelial cells playing pivotal roles in the repair process. Furthermore, prolonged exposure to PM2.5 exacerbates the risk of developing severe respiratory disorders, including lung injury, pulmonary fibrosis, asthma exacerbation, chronic obstructive pulmonary disease (COPD), and lung cancer. These chronic conditions significantly elevate the incidence and mortality related to respiratory diseases [12,13,14,15]. Epidemiological studies have confirmed that air pollution raises the incidence of lung cancer, and PM2.5 is an independent risk factor for lung cancer [16,17].

Long non-coding RNAs (lncRNAs), which are longer than 200 nucleotides, play diverse roles in cellular processes [18]. They participate in mRNA splicing, maturation, transport, localization, and stabilization [19]. Functioning as significant multi-dimensional modifiers, lncRNAs contribute extensively to translation regulation, post-transcriptional modifications, and epigenetic modulation [19]. A growing body of research suggests that lncRNAs play a role in critical biological processes associated with various pulmonary diseases [20,21]. *lncRNA PCAT1* activates *SOX2* to inhibit cGAS/STING signaling-mediated T-cell activation and promote tumorigenesis and immunosuppression [22]. *lncRNA H19* alleviates sepsis-induced acute lung injury by downregulating the expression of TNF-α, IL-6, IL-17, caspase-3, caspase-9, and Bax while upregulating Bcl-2 levels, thereby suppressing lung cell apoptosis and inflammation [23]. Studies have demonstrated that predicting lncRNA functions using co-expression networks of lncRNAs and mRNAs is advantageous for advancing research on PM2.5-induced lung injury [24,25].

Weighted gene co-expression network analysis (WGCNA) is an innovative algorithm designed to identify modules of co-expressed genes from gene expression profiling data [26]. This method elucidates the interactive mechanisms of genes, including hub lncRNAs and mRNAs, and establishes correlations among highly co-expressed modules. Despite its application in elucidating genes interactions in lung cancer [27,28,29], there is a notable absence of studies employing WGCNA to construct lncRNA–mRNA co-expression networks for investigating lung injury. Our study aims to investigate lncRNA–mRNA interactions in lung injury induced by PM2.5. The key lncRNAs and mRNAs were identified from Gene Expression Omnibus (GEO) (GSE138870). Employing WGCNA, we extracted modules of co-expressed lncRNAs and mRNAs and predicted their targeted interactions. Following the construction of the lncRNA–mRNA network, we explored lncRNA regulation of mRNAs in pathways. This analysis is expected to enhance our knowledge of lncRNA functions and their potential therapeutic implications in lung injury.

## 2. Results

### 2.1. Weighted Co-Expression Network Construction and Key Module Identification

**Figure 1:**
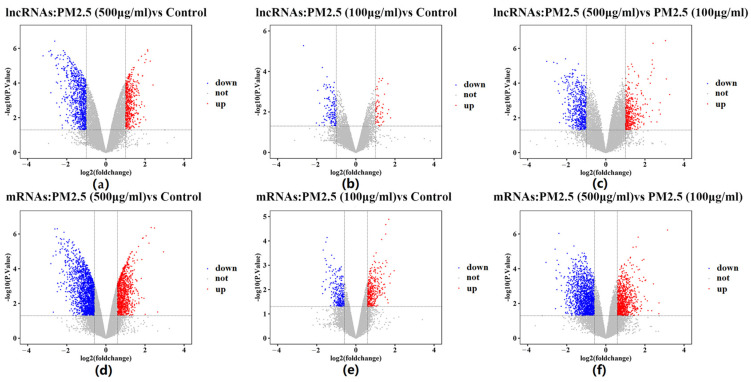
Differential expression analysis of lncRNAs and mRNAs under different PM2.5 exposure conditions. (**a**) lncRNAs: PM2.5 (500 μg/mL) vs. control; (**b**) lncRNAs: PM2.5 (100 μg/mL) vs. control; (**c**) lncRNAs: PM2.5 (500 μg/mL) vs. PM2.5 (100 μg/mL); (**d**) mRNAs: PM2.5 (500 μg/mL) vs. control; (**e**) mRNAs: PM2.5 (100 μg/mL) vs. control; (**f**) mRNAs: PM2.5 (500 μg/mL) vs. PM2.5 (100 μg/mL).

**Figure 2:**
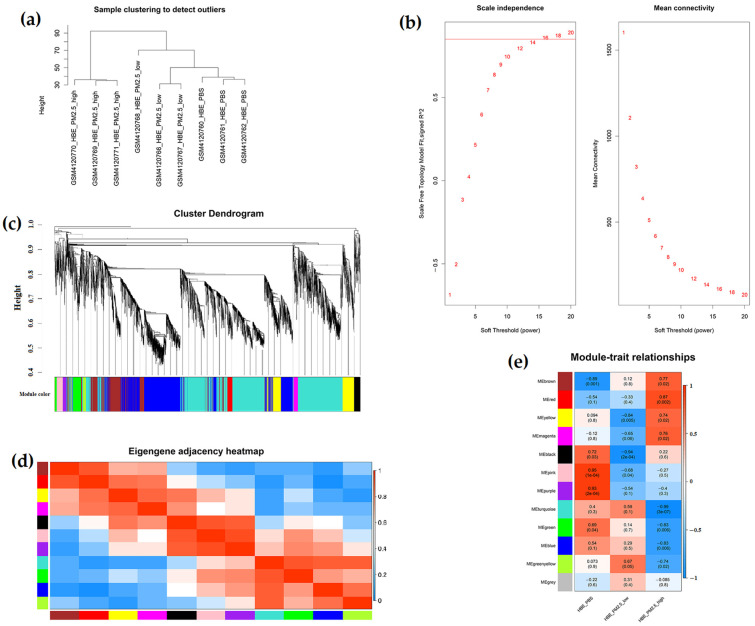
Identification of co-expression modules in different expression genes by WGCNA. (**a**) no outlier samples were found by cut-off height; (**b**) the soft threshold power of WGCNA; (**c**) the cluster dendrogram of WGCNA; (**d**) heatmap showing the adjacency matrix of eigengenes of various gene modules; (**e**) the correlation between modularity genes and lung injury.

Analysis of nine samples from the GSE138870 dataset revealed 1773 differentially expressed lncRNAs with fold change > 2 and 3381 mRNAs with fold change > 1.5 (Figure 1). The cutoff for lncRNAs was set at fold change > 2 to focus on the most robustly differentially expressed lncRNAs, while a slightly lower cutoff of fold change > 1.5 was used for mRNAs to capture a broader range of differentially expressed genes. These thresholds were chosen to ensure the most effective use of the data, balancing sensitivity and specificity. To examine the expression profiles of all lncRNAs and mRNAs, hierarchical clustering was performed to identify potential outlier samples. The results showed that there was no outlier sample (Figure 2a). Then, hierarchical clustering dendrograms were constructed to identify various modules (Figure 2b). Analysis of scale independence and mean connectivity resulted in the determination of a soft threshold power (β) value of 16 (Figure 2c). We obtained 12 modules with different expression trends (turquoise, blue, brown, yellow, green, red, black, pink, magenta, purple, green-yellow, and grey) (Table 1). The module relationships were visualized using a eigengene adjacency heatmap cluster (Figure 2d).

### 2.2. Correlation Analysis of Module and Traits

Figure 2e illustrates the association between co-expression modules and PM2.5 interventions. The red module demonstrated the highest positive correlation, with 500 μg/mL PM2.5 (correlation coefficient = 0.87, *p*-value = 0.002), while the turquoise module displayed the strongest negative correlation (correlation coefficient = −0.99, *p*-value = 3 × 10^7^). Moreover, we observed that green-yellow module exhibited the strongest positive correlation, with 100 μg/mL PM2.5 (correlation coefficient = 0.67, *p*-value = 0.05), while the black module showed the most pronounced negative correlation (correlation coefficient = −0.94, *p*-value = 2 × 10^4^). Based on the correlation between the modular genes and lung injury, as well as the number of lncRNAs and mRNAs, the turquoise, blue, brown, yellow, and green modules were selected for further analysis.

### 2.3. GO Enrichment Analysis and Pathway Enrichment Analysis

**Figure 3:**
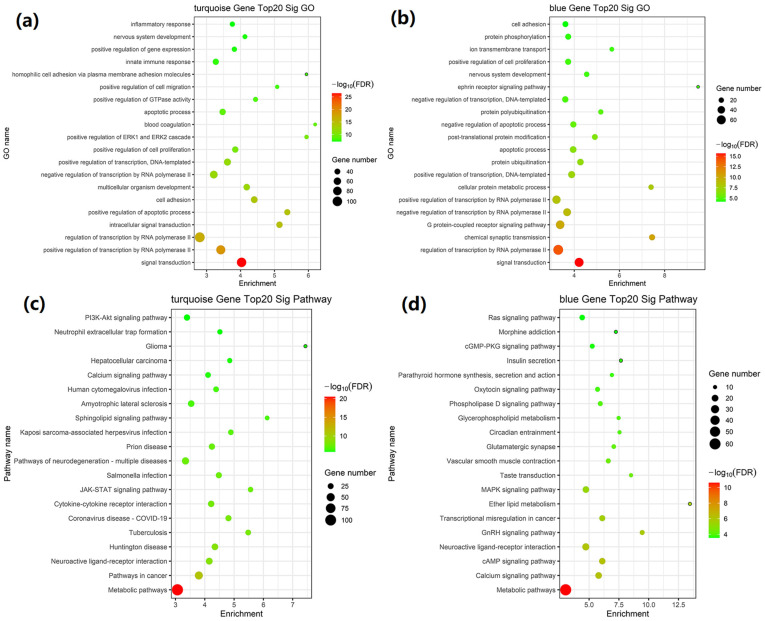
Functional enrichment analysis in the turquoise and blue modules. (**a**,**b**) Enriched GO analysis of mRNAs in turquoise and blue modules. (**c**,**d**) Enriched KEGG pathway analysis of mRNAs in turquoise and blue modules.

The GO enrichment analysis showed that the differentially expressed genes in the turquoise module were mainly enriched in signal transduction, positive regulation of transcription by RNA polymerase II, regulation of transcription by RNA polymerase II, intracellular signal transduction, and positive regulation of the apoptotic process (Figure 3a and Table 2). The results of GO enrichment analysis in blue modules were mainly enriched in signal transduction, regulation of transcription by RNA polymerase II, chemical synaptic transmission, G protein-coupled receptor signaling pathway, and negative regulation of transcription by RNA polymerase II (Figure 3b and Table 2). The GO enrichment of brown, yellow, and green modules is shown in Supplementary Figure S1.

KEGG pathway enrichment analysis revealed that the differentially expressed genes were primarily involved in metabolic pathways, pathways in cancer, neuroactive ligand–receptor interaction, Huntington disease, and tuberculosis in the turquoise module (Figure 3c and Table 3). The results of pathway enrichment in the blue modules were mainly related to metabolic pathways, calcium signaling pathway, cAMP signaling pathway, neuroactive ligand–receptor interaction, GnRH signaling pathway (Figure 3d and Table 3). The pathway enrichment of the brown, yellow, and green modules is shown in Supplementary Figure S1.

### 2.4. Construction of lncRNA–mRNA Co-Expression Networks

**Figure 4:**
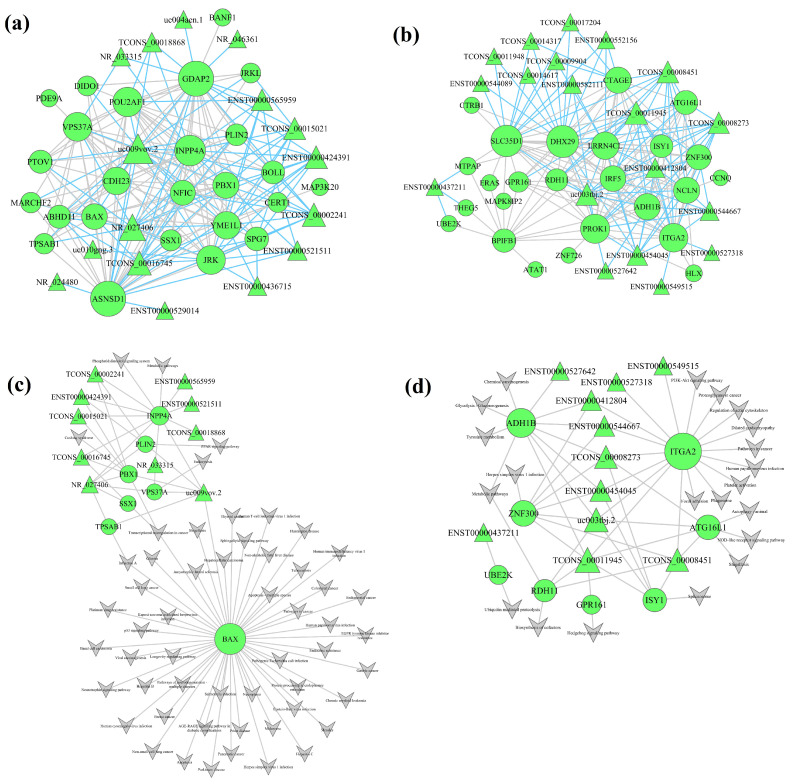
Module lncRNA–mRNA net of hub genes in the turquoise (**a**) and blue (**b**) modules. Module lncRNA–mRNA pathway net of hub genes in the turquoise (**c**) and blue (**d**) modules. Circles represent mRNAs, triangles represent lncRNAs, and gray polygons represent pathways. The size of the graph represents the level of intramodular connectivity of hub genes in the network.

lncRNA–mRNA co-expression networks were constructed for the functional prediction of lncRNA in the various modules (Figure 4). In the turquoise module, 25 mRNAs and 16 lncRNAs were identified as hub genes (Figure 4a), and 25 mRNAs and 19 lncRNAs as hub genes were detected in blue module (Figure 4b). Hub genes in the brown, yellow, and green modules are presented in Supplementary Figure S2. Previous research has shown that lncRNAs play an important role in disease development [30]. We found that several lncRNAs were closely associated with hub mRNAs in the co-expression network. It can be predicted that the lncRNA of unknown function at one end of the line has a similar function as the gene at the other end of the line. Hence, we can make inferences about the functions of lncRNAs on the basis of the functions of mRNAs.

### 2.5. Construction of lncRNA–mRNA-Pathway Network

To identify potential mechanisms of lncRNA-mediated regulation of signaling pathways, we constructed the lncRNA-mRNA-pathway network through interaction of significantly different pathways and lncRNA–mRNA co-expression networks (Figure 4 and Supplementary Figure S2). In the turquoise module, there were 10 lncRNAs and 7 mRNAs in the pathway network (Figure 4c). *NR_027406* (Alias: *LOC100129034*, a lncRNA) was linked to three mRNAs (*INPP4A*, *PBX1*, *VPS37A*, *TPSAB1*, *SSX1*, and *PLIN2*) and enriched in metabolic pathways, phosphatidylinositol signaling system, Cushing syndrome, transcriptional mis-regulation in cancer, endocytosis, Influenza A, transcriptional mis-regulation in cancer, and PPAR signaling pathway. *uc009vov.2* (Alias: *CROCCP2*, a lncRNA) was related to six mRNAs (*INPP4A*, *PBX1*, *PLIN2*, *VPS37A*, *BAX*, and *TPSAB1*) and abundant in 54 signaling pathways, such as metabolic pathways, transcriptional mis-regulation in cancer, PPAR signaling pathway, endocytosis, pathways in cancer, apoptosis, non-small-cell lung cancer, EGFR tyrosine kinase inhibitor resistance, non-alcoholic fatty liver disease, endocrine resistance, longevity-regulating pathway, apoptosis—multiple species, small-cell lung cancer, p53 signaling pathway, protein processing in endoplasmic reticulum, and basal cell carcinoma.

In the pathway network of the blue module (Figure 4d), *TCONS_00011945* (Alias: *lnc-CLVS2-2*, a lncRNA) was linked to seven mRNAs (*ITGA2*, *ADH1B*, *ATG16L1*, *ISY1*, *ZNF300*, *GPR161*, and *RDH11*) and enriched in 20 signaling pathways, such as platelet activation, pathways in cancer, proteoglycans in cancer, focal adhesion, PI3K-Akt signaling pathway, metabolic pathways, glycolysis/gluconeogenesis, chemical carcinogenesis, tyrosine metabolism, autophagy—animal, and NOD-like receptor signaling pathway. *uc003tbj.2* (Alias: *GARS1-DT*, a lncRNA) was also linked to six mRNAs (*ITGA2*, *ADH1B*, *ZNF300*, *RDH11*, *ISY1*, and *ATG16L1*) and abundant in 19 signaling pathways, such as platelet activation, pathways in cancer, proteoglycans in cancer, focal adhesion, phagosome, human papillomavirus infection, PI3K-Akt signaling pathway, dilated cardiomyopathy, regulation of actin cytoskeleton, and metabolic pathways.

### 2.6. Construction of Protein–Protein Interaction Network

**Figure 5:**
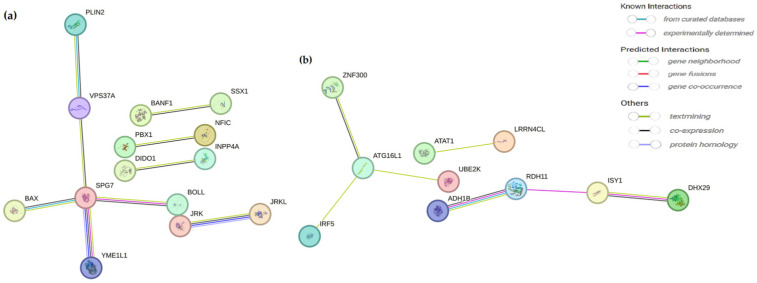
Construction of protein–protein interaction (PPI) networks in the turquoise (**a**) and blue (**b**) modules.

To investigate the interactions among hub genes within each module, we used the STRING database to construct protein–protein interaction (PPI) networks (Figure 5). The PPI network for the turquoise module is shown in Figure 5a, comprising 14 key genes identified through PPI analysis. Similarly, the PPI network for the blue module is presented in Figure 5b, with 10 crucial genes identified within this module based on the PPI analysis.

## 3. Discussion

Recent studies have provided growing evidence that exposure to PM2.5 is associated with a significant increase in lung injury and a decline in lung function [31]. Genomic studies have identified several genes implicated in PM2.5-induced lung injury [14,32,33]. However, the underlying mechanisms of PM2.5-induced lung injury remain unclear. WGCNA was applied for the first time to explore the relationship between PM2.5 exposure and lung cell injury. Using the GEO database (GSE138870 dataset), we identified a total of 3391 mRNAs and 1773 lncRNAs. Through WGCNA analysis, we categorized these lncRNAs and mRNAs into 12 modules with diverse functionalities, revealing five modules (turquoise, blue, brown, yellow, and green) closely associated with lung injury. Additionally, functional enrichment analysis was performed to investigate the potential biological impacts of lncRNAs and mRNAs in lung cell injury. The lncRNA–mRNA co-expression network provided novel insights into the interplay between lncRNAs and mRNAs. Furthermore, we constructed a lncRNA–mRNA-pathway co-expression network and identified positive associations with metabolic pathways, calcium signaling pathway, pathways in cancer, cAMP signaling pathway, MAPK signaling pathway, and other signaling pathways. These findings suggest that lung cell injuries may arise from disruptions in multiple signaling pathways, underscoring the regulatory role of PM2.5 in lung cell injury.

In contrast to the identification of differentially expressed genes, WGCNA utilizes large-scale gene expression data to delineate co-expression modules and conduct phenotype association studies [34]. Hub genes within WGCNA modules play significant regulatory roles in various biological processes [35,36]. Consequently, investigating hub genes in modules becomes increasingly crucial for elucidating the molecular mechanisms underlying lung cell injury. In our study, we especially examined the hub genes within the turquoise and blue modules. Within these modules, numerous hub mRNAs were explored to positively regulate critical biological processes, such as metabolism, cell cycle regulation, apoptosis, oxidative stress response, and inflammation. These findings suggest that perturbations in metabolic pathways, calcium signaling pathway, pathways in cancer, cAMP signaling pathway, and MAPK signaling pathway, which are mediated by the concerted actions of multiple genes, may contribute to the pathogenesis of lung cell injury.

LncRNAs can interact with proteins, RNA, and even DNA, mediating gene regulation by acting as signals to stimulate or inhibit transcription processes. As lncRNAs post-transcriptionally regulate mRNAs, predicting their interactions and exploring related mechanisms in pathological processes through regulatory networks is essential [19]. Jiang et al. [37] characterized the lncRNA–mRNA regulatory networks in PM2.5-induced lung cancer with expression profile data and identified the potential therapeutic target, the *LCPAT1*-*RCC2* axis. Furthermore, PM2.5 can be internalized by lung cancer cells, resulting in significant increases in reactive oxygen species (ROS) levels, along with upregulation of *loc146880* and *LC3B* expression, thereby promoting autophagy [38]. In the present study, multiple novel PM2.5-regulated lncRNA–mRNA networks were identified, providing valuable insights into the mechanisms underlying PM2.5 exposure and lung cell injury.

In the turquoise module, *LOC100129034*, *CROCCP2*, *ENST00000424391* (Alias: *MYO3B-AS1*), *TCONS_00002241* (Alias: *linc-SRP9-1*), and *TCONS_00016745* were key lncRNAs linked to hub mRNAs (*BAX*, *INPP4A*, *PBX1*, *PLIN2*, *VPS37A*, *SSX1*, and TPSAB1). *BAX* (BCL2 associated X) plays a pivotal role as an effector molecule in the process of mitochondria-dependent programed cell death [39,40]. An in vivo study showed that PM2.5 significantly increased the expression of BAX and induced mitochondrial damage and cell apoptosis [32] to enhance lung injury [41]. In sepsis-induced acute lung injury, upregulation of *lncRNA H19* inhibited TNF-α, IL-1β, IL-6, IL-10, and BAX to suppress pulmonary apoptosis and inflammation [23,42]. In addition, Xu et al. reported that *LINC01089* interacted with the *miR-543*/*BAX* axis to inhibit lung adenocarcinoma cell proliferation and promoted apoptosis [43]. Elevated levels of *lncRNA GHRLOS* significantly inhibited cancer cell proliferation and invasion while promoting apoptosis through the modulation of E-cadherin, N-cadherin, BAX, and Bcl-2 expression in non-small-cell lung cancer [44]. Therefore, existing evidence suggests that *INPP4A* is correlated with airway inflammation and remodeling and plays a crucial role in maintaining lung homeostasis [45,46]. It has been demonstrated previously that the other hub mRNAs (*PBX1*, *PLIN2*, and *TPSAB1*) are closely related to the progression of lung cancer and COPD by regulating the cell cycle and cell proliferation [47,48,49]. Consequently, we postulated that the key lncRNAs, *LOC100129034*, *CROCCP2*, *MYO3B-AS1*, *linc-SRP9-1*, and *TCONS_00016745*, which were co-expressed with *BAX*, *INPP4A*, *PBX1*, *PLIN2*, *VPS37A*, *SSX1*, and *TPSAB1* in our WGCNA analysis, might play a role in apoptosis and proliferation modulation during PM2.5-induced lung cell injury.

In the blue module, *lnc-CLVS2-2* and *GARS1-DT* were the core lncRNAs connected to hub genes (*ITGA2*, *ADH1B*, *ATG16L1*, *ISY1*, *ZNF300*, *GPR161*, and *RDH11*). Recent results have indicated that *ITGA2* activated the FAK-RAC1-PAK signaling pathway to participate in the formation of the cytoskeleton in lung adenocarcinoma cells and then promoted migration and invasion [50]. In the process of methotrexate-induced adverse reactions, such as serious lung injury, *ITGA2* plays a key role in the epithelial–mesenchymal transition (EMT) [51]. The ATG16L1 protein was found to be associated with lung injury and inflammation levels in mice with Pseudomonas aeruginosa lung infection and LPS-induced acute lung injury [52,53]. Additionally, *ADH1B*, *ZNF300*, *GPR161*, and *RDH11* were presented as the novel regulatory genes in lung injury and lung cancer [54,55,56,57]. We predict that *lnc-CLVS2-2* and *GARS1-DT* might interact with *ITGA2*, *ADH1B*, *ATG16L1*, *ISY1*, *ZNF300*, *GPR161*, and *RDH11* in the process of PM2.5-induced lung cell injury through the pathways in cancer, proteoglycans in cancer, focal adhesion, PI3K-Akt signaling pathway, metabolic pathways, chemical carcinogenesis, and PI3K-Akt signaling pathways.

## 4. Materials and Methods

### 4.1. Data Retrieving and Processing

**Figure 6:**
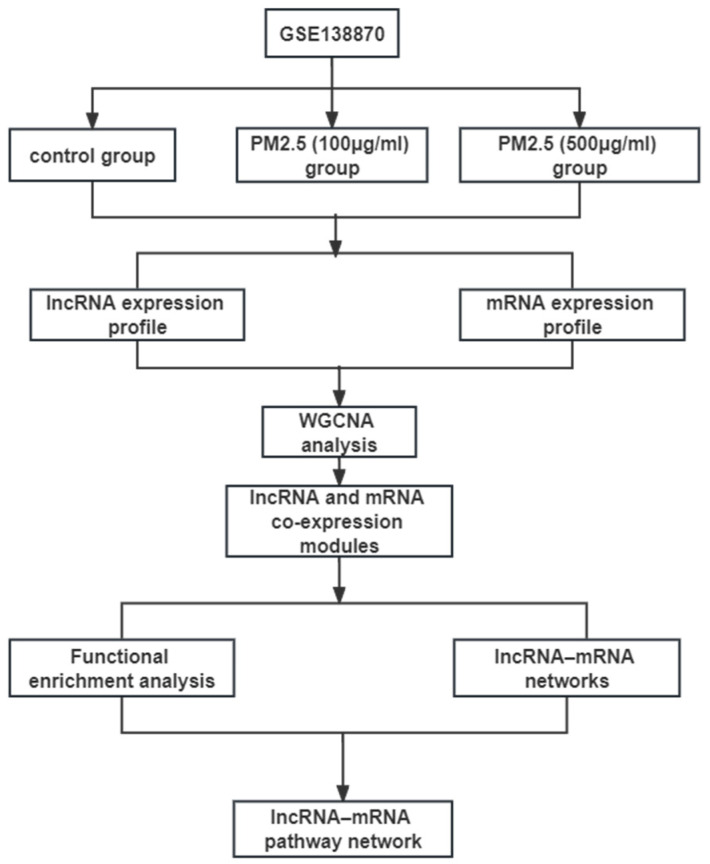
The main procedure of the study. WGCNA, weighted correlation network analysis.

The expression profiling of GSE138870, which is identified on the GPL16956 platform (Agilent-045997 Arraystar human lncRNA microarray V3 (Probe Name Version)), was downloaded from the GEO database (https://www.ncbi.nlm.nih.gov/geo/query/acc.cgi?acc=GSE138870, accessed on 14 March 2023). GSE138870 contained nine HBE cell samples from three normal cells, three HBE cells exposed to 100 μg/mL PM2.5, and three HBE cells exposed to 500 μg/mL PM2.5. HIST2 and Stringtie were used to re-align the human standard genome and gene annotation [58]. Significant differentially expressed lncRNAs and mRNAs between the control and exposure groups were screened by R software (version 4.1.1) with the screening criteria (fold change > 2 or >1.5, *p* < 0.05, false discovery rate (FDR) < 0.05). The flow chart of this research is shown in Figure 6.

### 4.2. Weighted Gene Co-Expression Network Analysis (WGCNA)

WGCNA is used to find highly co-expressed gene modules and associations between genomes and diseases [59]. We used the R package “WGCNA” to evaluate the availability of genes and construct the gene co-expression network. The Person coefficient was calculated for the strength of correlation between any two genes [60]. Hierarchical cluster trees were created based on the correlation coefficients between genes, with distinct branches representing different gene modules. Genes were classified according to their expression patterns using weighted correlation coefficients, and those with similar patterns were grouped into modules.

### 4.3. Gene Ontology and Pathway Enrichment Analysis

The functional annotation of mRNAs in each module was analyzed using the Gene Ontology (GO) database. The Kyoto Encyclopedia of Genes and Genomes (KEGG) database is a public pathway database to perform pathway enrichment analysis. Fisher’s exact test and multiple comparison tests were employed to calculate the *p*-value and FDR. *p* < 0.05 and FDR < 0.05 indicated significant differences.

### 4.4. Construction of lncRNA–mRNA Co-Expression Networks

The hub genes were defined by higher connectivity in the module. Hub genes with high connectivity were upstream regulators, while low-connectivity genes were downstream regulators. Co-expression relationships between hub genes were calculated, and lncRNA–mRNA co-expression and mRNA–mRNA co-expression were selected to construct a co-expression network.

### 4.5. Construction of lncRNA–mRNA-Pathway Co-Expression Network

In order to construct an lncRNA–mRNA pathway net, the regulatory relationship between lncRNAs and mRNAs was detected, as well as the significant pathways involved in mRNA regulation. The primary objective of this study was to reveal lncRNA-regulated signaling pathways to predict potential diseases associated with lncRNAs.

### 4.6. Protein–Protein Interaction (PPI) Network Construction

PPI network analysis was conducted using the online tool STRING (version 11.5; https://cn.string-db.org/, accessed on 27 December 2024) [61]. In our study, both lncRNAs and mRNAs within the identified modules were subjected to analysis using STRING.

### 4.7. Statistical Analysis

Statistical analysis was conducted using R software (Version 4.1.1) and SPSS (Version 21.0). Statistical comparisons between groups of normalized data were performed with Student’s *t* test or one-way analysis of variance. *p* < 0.05 was considered to be significant.

## 5. Conclusions

In conclusion, based on WGCNA, we identified PM2.5-induced lung cell injury-related candidate hub lncRNAs and mRNAs, and we constructed both lncRNA–mRNA co-expression networks and lncRNA–mRNA pathway co-expression networks. The results indicated that the hub lncRNAs may regulate the inflammatory, metabolic, and cancer pathways, which are critically involved in the pathogenesis of lung cell injury. Moreover, WGCNA analysis showed potential as a novel predictive tool for PM2.5-induced lung cell injury. Therefore, future research should focus on investigating the regulatory roles of hub lncRNAs and mRNAs in PM2.5-induced lung cell injury.

## Figures and Tables

**Table 1:** The numbers of lncRNAs and mRNAs in the 12 modules.

Module	All Numbers	lncRNA Numbers	mRNA Numbers
turquoise	2221	764	1457
blue	1365	519	846
brown	616	239	377
yellow	404	161	243
green	186	42	144
red	129	48	81
black	112	30	82
pink	101	41	60
magenta	75	15	60
purple	65	21	44
green-yellow	50	18	32
grey	2	0	2

**Table 2:** Top 20 significantly changed GOs of differentially expressed genes in turquoise and blue modules.

Module	GO id	Term	No. of Genes	−logP
turquoise module	GO:0007165	signal transduction	98	30.03
	GO:0045944	positive regulation of transcription by RNA polymerase II	89	22.21
	GO:0006357	regulation of transcription by RNA polymerase II	106	19.79
	GO:0035556	intracellular signal transduction	45	17.80
	GO:0043065	positive regulation of apoptotic process	42	17.33
	GO:0007155	cell adhesion	50	16.87
	GO:0007275	multicellular organism development	47	14.99
	GO:0000122	negative regulation of transcription by RNA polymerase II	64	14.64
	GO:0045893	positive regulation of transcription, DNA-templated	54	14.46
	GO:0008284	positive regulation of cell proliferation	44	12.73
	GO:0070374	positive regulation of ERK1 and ERK2 cascade	28	12.69
	GO:0007596	blood coagulation	25	11.76
	GO:0006915	apoptotic process	45	11.47
	GO:0043547	positive regulation of GTPase activity	32	10.94
	GO:0030335	positive regulation of cell migration	27	10.59
	GO:0007156	homophilic cell adhesion via plasma membrane adhesion molecules	23	10.44
	GO:0045087	innate immune response	43	10.11
	GO:0010628	positive regulation of gene expression	34	9.78
	GO:0007399	nervous system development	31	9.78
	GO:0006954	inflammatory response	34	9.60
blue module	GO:0007165	signal transduction	60	19.18
	GO:0006357	regulation of transcription by RNA polymerase II	73	17.32
	GO:0007268	chemical synaptic transmission	25	13.37
	GO:0007186	G protein-coupled receptor signaling pathway	53	12.95
	GO:0000122	negative regulation of transcription by RNA polymerase II	43	11.74
	GO:0045944	positive regulation of transcription by RNA polymerase II	49	11.20
	GO:0044267	cellular protein metabolic process	20	10.66
	GO:0045893	positive regulation of transcription, DNA-templated	34	9.90
	GO:0016567	protein ubiquitination	29	9.41
	GO:0006915	apoptotic process	30	8.92
	GO:0043687	post-translational protein modification	23	8.63
	GO:0043066	negative regulation of apoptotic process	26	7.75
	GO:0000209	protein polyubiquitination	19	7.50
	GO:0045892	negative regulation of transcription, DNA-templated	27	7.19
	GO:0048013	ephrin receptor signaling pathway	11	7.01
	GO:0007399	nervous system development	20	6.95
	GO:0008284	positive regulation of cell proliferation	25	6.95
	GO:0034220	ion transmembrane transport	16	6.87
	GO:0006468	protein phosphorylation	24	6.68
	GO:0007155	cell adhesion	24	6.41

**Table 3:** Top 20 significantly changed pathways of differentially expressed genes in turquoise and blue modules.

Module	Pathway ID	Term	No. of Genes	−logP
turquoise module	01100	Metabolic pathways	107	23.01
	05200	Pathways in cancer	47	13.39
	04080	Neuroactive ligand–receptor interaction	33	10.47
	05016	Huntington disease	31	10.35
	05152	Tuberculosis	23	9.71
	05171	Coronavirus disease—COVID-19	26	9.66
	04060	Cytokine-cytokine receptor interaction	29	9.37
	04630	JAK-STAT signaling pathway	21	8.99
	05132	Salmonella infection	26	8.98
	05022	Pathways of neurodegeneration—multiple diseases	37	8.98
	05020	Prion disease	27	8.79
	05167	Kaposi sarcoma-associated herpesvirus infection	22	8.33
	04071	Sphingolipid signaling pathway	17	7.95
	05014	Amyotrophic lateral sclerosis	30	7.86
	05163	Human cytomegalovirus infection	23	7.78
	04020	Calcium signaling pathway	23	7.25
	05225	Hepatocellular carcinoma	19	7.15
	05214	Glioma	13	7.12
	04613	Neutrophil extracellular trap formation	20	6.99
	04151	PI3K-Akt signaling pathway	28	6.96
blue module	01100	Metabolic pathways	62	13.08
	04020	Calcium signaling pathway	19	8.32
	04024	cAMP signaling pathway	18	8.24
	04080	Neuroactive ligand–receptor interaction	22	7.94
	04912	GnRH signaling pathway	12	7.63
	05202	Transcriptional misregulation in cancer	16	7.33
	00565	Ether lipid metabolism	9	7.10
	04010	MAPK signaling pathway	19	6.88
	04742	Taste transduction	10	5.93
	04270	Vascular smooth muscle contraction	12	5.88
	04724	Glutamatergic synapse	11	5.68
	04713	Circadian entrainment	10	5.43
	00564	Glycerophospholipid metabolism	10	5.39
	04072	Phospholipase D signaling pathway	12	5.38
	04921	Oxytocin signaling pathway	12	5.20
	04928	Parathyroid hormone synthesis, secretion, and action	10	5.08
	04911	Insulin secretion	9	4.94
	04022	cGMP-PKG signaling pathway	12	4.83
	05032	Morphine addiction	9	4.74
	04014	Ras signaling pathway	14	4.74

## Data Availability

Data are available in a publicly accessible repository.

## Supplementary Materials

The following supporting information can be downloaded at: https://www.mdpi.com/article/10.3390/ijms26030911/s1.

## Abbreviations

The following abbreviations are used in this manuscript:

PM2.5	Fine particulate matter
WGCNA	Weighted gene co-expression network analysis
GEO	Gene Expression Omnibus
COPD	Chronic obstructive pulmonary disease
lncRNAs	Long non-coding RNAs
ROS	Reactive oxygen species
FDR	False discovery rate
