# Supplementary material for: Identification and Evaluation of Hub Long Non-Coding RNAs and mRNAs in PM2.5-Induced Lung Cell Injury
Source: Int J Mol Sci. 2025 Jan 22;26(3):911. doi: 10.3390/ijms26030911 (PMC11816485; doi:10.3390/ijms26030911)
Supplement: Supplementary file 1 [file ijms-26-00911-s001.zip › ijms-3430199-supplementary.pdf]

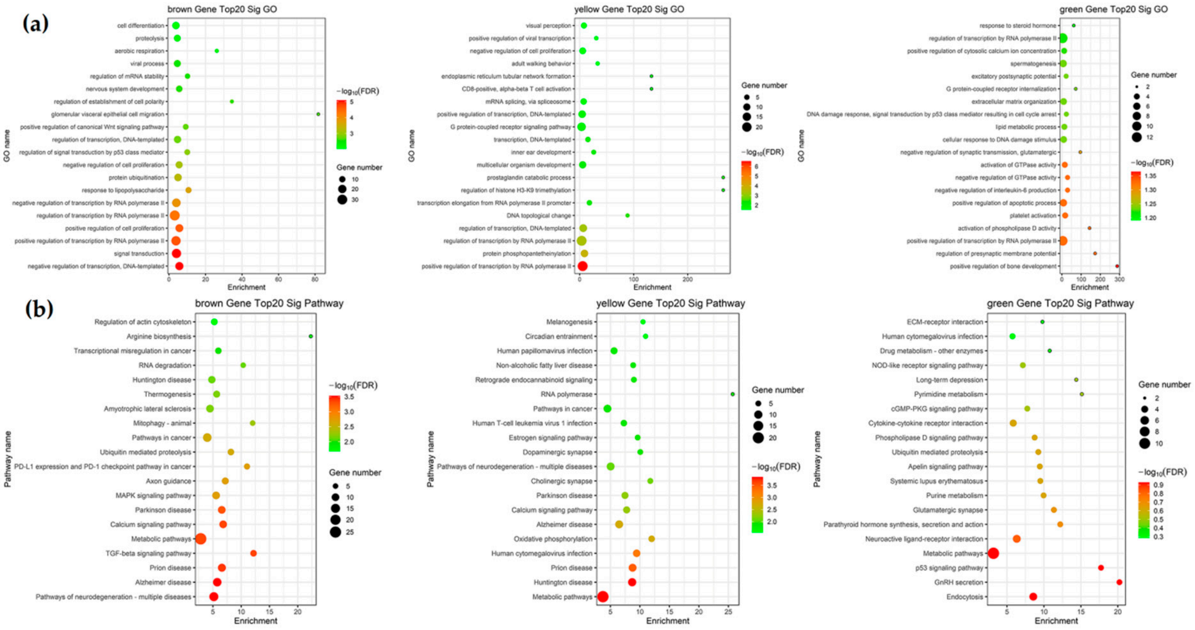

Figure S1. Functional enrichment analysis in the brown, yellow, and green modules. (a) Enriched GO analysis of mRNAs; (b) Enriched KEGG pathway analysis of mRNAs.

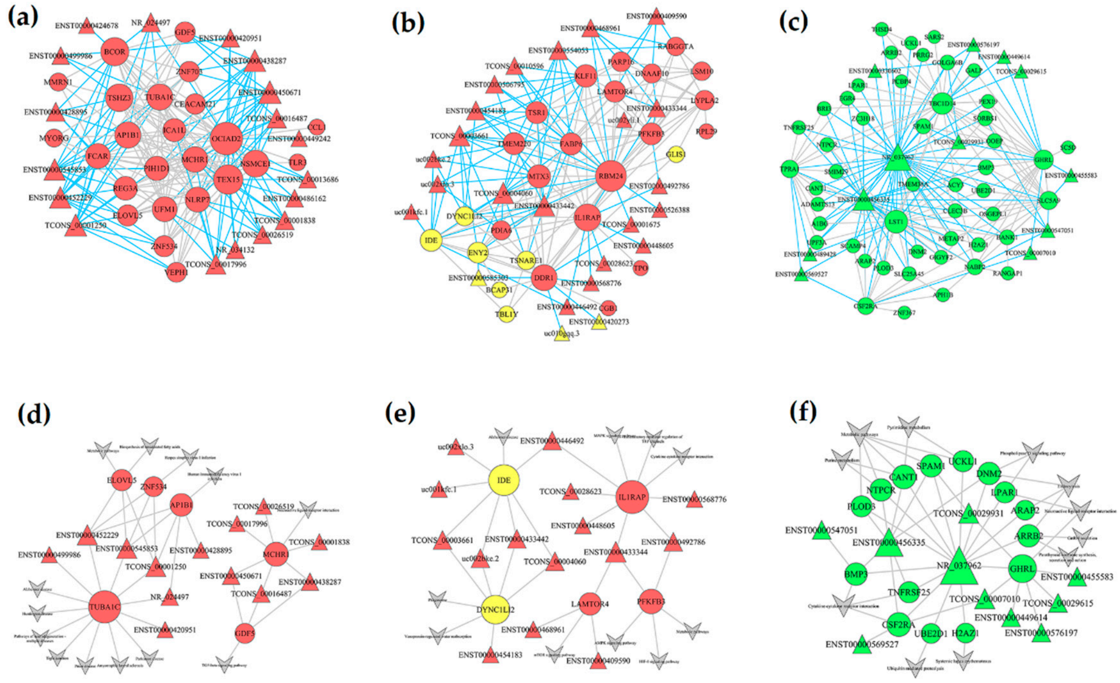

Figure S2. Module lncRNA–mRNA net of hub genes in the brown (a), yellow (b), and green (c) modules. Module lncRNA–mRNA pathway net of hub genes in the brown (d), yellow (e), and green (f) modules. Circles represent mRNAs, triangles represent lncRNAs, and gray polygons repre-sent pathways. Red represents upregulation, green represents down-regulation, yellow represents both up-and down-regulation (because pairwise comparisons are three groups). The size of the graphs represents the level of intramodular connectivity of hub genes in the network.
